# Cerebrospinal fluid and serum proteomic profiles accurately distinguish neuroaxonal dystrophy from cervical vertebral compressive myelopathy in horses

**DOI:** 10.1111/jvim.16660

**Published:** 2023-03-16

**Authors:** Callum G. Donnelly, Amy L. Johnson, Steve Reed, Carrie J. Finno

**Affiliations:** ^1^ Department of Population Health and Reproduction, School of Veterinary Medicine University of California, Davis Davis California USA; ^2^ Department of Clinical Studies, New Bolton Center, School of Veterinary Medicine University of Pennsylvania Kennett Square Pennsylvania USA; ^3^ Rood and Riddle Equine Hospital Lexington Kentucky USA

**Keywords:** biomarker, machine learning, neurodegeneration, precision medicine

## Abstract

**Background:**

Cervical vertebral compressive myelopathy (CVCM) and equine neuroaxonal dystrophy/degenerative myeloencephalopathy (eNAD/EDM) are leading causes of spinal ataxia in horses. The conditions can be difficult to differentiate, and there is currently no diagnostic modality that offers a definitive antemortem diagnosis.

**Objective:**

Evaluate novel proteomic techniques and machine learning algorithms to predict biomarkers that can aid in the antemortem diagnosis of noninfectious spinal ataxia in horses.

**Animals:**

Banked serum and cerebrospinal fluid (CSF) samples from necropsy‐confirmed adult eNAD/EDM (n = 47) and CVCM (n = 25) horses and neurologically normal adult horses (n = 45).

**Methods:**

. A subset of serum and CSF samples from eNAD/EDM (n = 5) and normal (n = 5) horses was used to evaluate the proximity extension assay (PEA). All samples were assayed by PEA for 368 neurologically relevant proteins. Data were analyzed using machine learning algorithms to define potential diagnostic biomarkers.

**Results:**

Of the 368 proteins, 84 were detected in CSF and 146 in serum. Eighteen of 84 proteins in CSF and 30/146 in serum were differentially abundant among the 3 groups, after correction for multiple testing. Modeling indicated that a 2‐protein test using CSF had the highest accuracy for discriminating among all 3 groups. Cerebrospinal fluid R‐spondin 1 (RSPO1) and neurofilament‐light (NEFL), in parallel, predicted normal horses with an accuracy of 87.18%, CVCM with 84.62%, and eNAD/EDM with 73.5%.

**Main Limitations:**

Cross‐species platform. Uneven sample size.

**Conclusions and Clinical Importance:**

Proximity extension assay technology allows for rapid screening of equine biologic matrices for potential protein biomarkers. Machine learning analysis allows for unbiased selection of highly accurate biomarkers from high‐dimensional data.

**Abbreviations**
CALB2: calretinin
COV: coefficient of variation
CSF: cerebrospinal fluid
CVCM: cervical vertebral compressive myelopathy
DKK1: dikkoph‐1
eNAD/EDM: equine neuroaxonal dystrophy/degenerative myeloencephalopathy
NEFL: neurofilament‐light
NPX: normalized expression units
PCA: principal component analysis
PEA: proximity extension assay
pNfH: phosphorylated neurofilament heavy
RSPO1: r‐spondin 1
SOD2: superoxide dismutase‐2
WFKKN1: WAP, follistatin/kazal, immunoglobulin, kunitz and neutrin domain containing 1

## 1 INTRODUCTION

Spinal ataxia is a frequently encountered neurologic problem in racing and sport horses. Spinal ataxia has several underlying causes, including congenital and acquired cervical vertebral compressive myelopathy (CVCM), equine neuroaxonal dystrophy (eNAD)/equine degenerative myeloencephalopathy (EDM), and equine protozoal myeloencephalitis (EPM). Apart from EPM, definitively diagnosing the underlying cause of spinal ataxia antemortem is challenging.^1^ Equine protozoal myeloencephalitis is an infectious disease that reliably induces antibody production in horses, allowing immunologic confirmation of central nervous system (CNS) infection. Cervical vertebral compressive myelopathy and eNAD/EDM are noninfectious diseases that result in progressive neurodegeneration, with CVCM the result of structural vertebral column abnormalities and eNAD/EDM the result of genetic predilection and contributing factors such as inadequate vitamin E status. Physical aspects of the horse preclude sensitive imaging techniques such as magnetic resonance imaging (MRI), with diagnosis relying on radiography, myelography, computed tomography, clinical exclusion, or a combination of these. These imaging techniques are used primarily to exclude CVCM, but the poor sensitivity and presence of nonrelevant background lesions limit their utility.^2^ Additionally, no definitive diagnostic test is available for the second most common cause of spinal ataxia, eNAD/EDM.^3^, ^4^ As such, definitive diagnosis in many cases of spinal ataxia can only be achieved by necropsy. Inability to accurately diagnose spinal ataxia antemortem is an important financial burden for horse owners, trainers, and insurance underwriters.

Next‐generation proteomic technologies, coupled with detailed phenotyping, provide the opportunity to enhance diagnostic modalities. Spinal ataxia in horses is well‐positioned to benefit from these technologies and improve antemortem diagnosis. The application of such techniques to degenerative neurologic disease in humans already has yielded several highly sensitive and specific biomarkers.^5^ Of particular interest is the use of discovery proteomics to rapidly advance the availability of diagnostic and prognostic markers such as neurofilament light chain (NEFL). Neurofilament light chain is a sensitive and specific marker of neurodegeneration in humans, with serum and cerebrospinal fluid (CSF) concentrations increased years before the onset of symptoms.^6^ The potential to apply these same discovery approaches to spinal ataxia in horses is now possible. Therefore, our aim was to define the serum and CSF proteome of horses with CVCM and eNAD/EDM for a targeted set of proteins curated for neuropathologic conditions. Our primary hypothesis was that horses with spinal ataxia will have a unique proteomic profile in serum and CSF that distinguishes them from neurologically normal horses. We additionally hypothesized that CVCM and eNAD/EDM will have distinct proteomic profiles that differentiate each cause of spinal ataxia.

## 2 METHODS

### 2.1 Pilot study

Samples of CSF and serum from age‐ and sex‐matched American Quarter Horses were used to validate the Olink proximity extension assay technology (PEA). Samples were derived from necropsy‐confirmed eNAD (n = 5) and neurologically normal horses (n = 5). Samples were evaluated using the Olink Target 92 Neuro‐Exploratory assay. This assay simultaneously evaluates the relative concentration of 92 curated proteins in serum and CSF without the need for additional sample preparation. Olink selects proteins to encompass both potential biomarker targets and representative proteins of important biologic pathways. All assays were performed at Olink (Boston, Massachusetts), with samples run on a single plate. Internal plate standardization and quality control were performed by Olink.

### 2.2 Study cohort

Previously collected paired serum and CSF samples from neurologically normal horses (n = 45) formed the control cohort. Paired serum and CSF samples from CVCM (n = 25) and eNAD/EDM (n = 47) horses that all were examined for spinal ataxia and later confirmed by necropsy formed the case population*s*. All samples were collected as previously described^7^ and stored at −80°C until analysis. Samples used for the study had not previously undergone a freeze‐thaw cycle until the time of analysis.

Sample size was calculated according to the National Cancer Institute's guidelines for classifier development for high‐dimensional data.^8^, ^9^ Using a standard fold change of 2, with a protein array of 368 analytes and a training set sample tolerance = 0.1, at least 19 samples per group are required to accurately assign groups based on the array. Standard prevalence parameters were used because the prevalence of this group was known.

### 2.3 Proximity extension assay analysis

Study samples were quantified using Olink multiplex PEA panels (Olink Proteomics; www.olink.com) according to the manufacturer's instructions and as described previously.^10^ The basis of PEA is a dual‐recognition immunoassay, where 2 matched antibodies labeled with unique DNA oligonucleotides simultaneously bind to a target protein in solution. This process brings the 2 antibodies into proximity, allowing their DNA oligonucleotides to hybridize, serving as template for a DNA polymerase‐dependent extension step. This double‐stranded DNA, which is unique for specific antigens, is amplified using P5/P7 Illumina adapters along with sample indexing, which is quantitatively proportional to the initial concentration of target protein. Finally, amplified targets are quantified by Next Generation Sequencing using Illumina Nova Seq 6000 (Illumina Corporation, San Diego, California). We used the Explore 384 Neurology panel, which measures 368 proteins using 1 μL of serum and CSF.

### 2.4 Data analysis

Relative quantification of protein expression was calculated by read counts normalized to the internal plate controls, as described previously.^10^ Read counts then were converted to normalized protein expression units (NPX), providing relative quantification on a log_2_ scale.

Proteins were analyzed if they were detectable in ≥50% of samples. Analysis was performed using the OlinkAnalyze package in R (R project, San Diego, California). Data were first inspected by principal component analysis (PCA). Analysis of covariance (ANCOVA) was performed for serum and CSF samples, with phenotype (eNAD/EDM, CVCM and normal) considered a fixed effect and age, breed, and sex introduced as covariates. A Bonferroni correction was used to correct for multiple comparisons. A corrected *P*‐value of <.05 was considered significant.

Proteins that were significantly differentially abundant in serum and CSF were used to train random forest models. Random forest analysis was performed in R using the randomForest package with default parameters. A randomly selected subset of cases and controls (80%) was used to train the model. The model subsequently was tested on the remaining cases and controls (20%). Models were constructed for serum alone, CSF alone and a merged serum and CSF dataset. Results are reported as class error (proportion of incorrect decisions), mean decrease in accuracy and mean decrease in Gini index. The Gini index gives the probability of a feature (ie, protein marker), selected randomly, that is incorrectly classified.

To determine the minimum biomarker set needed to achieve accurate group assignment, conditional inference models were constructed in R using the Partykit package. Default parameters were used for the 3 classifiers, with the model having the same set of proteins introduced as the random forest model for serum, CSF and merged data. A *P*‐value of <.05 was used to define binary classification with accuracy and precision of prediction for each group reported.

## 3 RESULTS

### 3.1 Pilot study

All CSF samples and 9/10 serum samples fully passed quality control, with intra‐assay coefficients of variation of 3% and 8%, respectively. In serum, 36/92 proteins were detected in ≥50% samples and in CSF 15/92 proteins were detected in ≥50% of samples (Supplementary Table 1). Because of the small sample size, differential abundance analysis was not performed. Our preliminary data indicate that the Olink platform performs favorably with equine‐derived samples.

### 3.2 Serum proteins

Quality control parameters were met for 86% of individual assays, with an intra‐assay coefficient of variation (COV) of 12%. In >50% of samples, 148 of 368 proteins (40%) were detected. Of those proteins detected in >50% samples, 37 (25%) were differentially abundant among the 3 groups (Supplementary Table 2). Despite the relatively large number of differentially abundant proteins identified, the PCA for serum did not indicate clear clustering by group (Supplementary Figure 1).

### 3.3 CSF proteins

**FIGURE 1:**
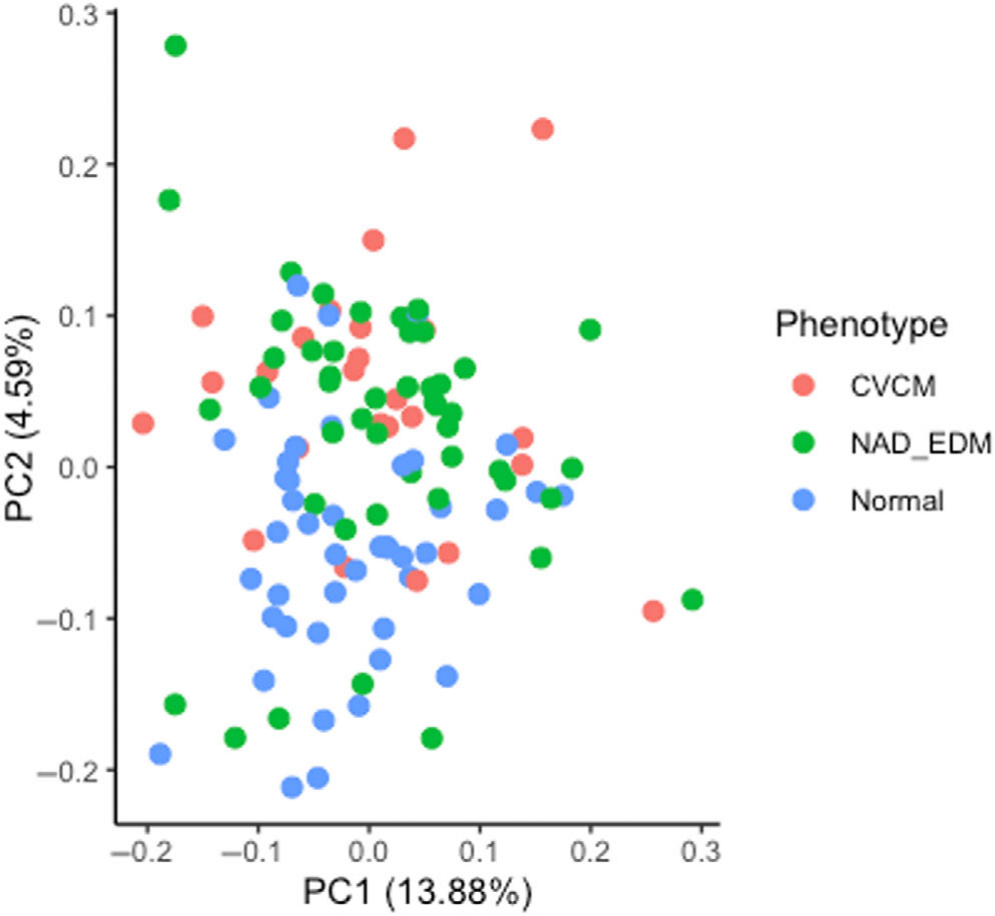
Principal component plot of CSF proteins showing the first two components (PC1 and PC2). Using proteins with a missingness ≤50%, values were converted to eigenvectors and plotted. Each dot represents an individual horse from the CVCM group (n = 25; salmon), eNAD/EDM (n = 45; green), or normal (n = 43; blue).

Quality control parameters were met for 89% of individual assays, with an intra‐assay‐COV of 12%. In >50% of samples, 84 of 368 proteins (23%) were detected. Of those proteins detected in >50% samples, 18 (21%) were differentially abundant among the 3 groups (Supplementary Table 3). The PCA analysis indicated a clear distinction between samples derived from animals with spinal ataxia compared with neurologically normal horses (Figure 1). However, a high degree of overlap was observed between the 2 groups of horses with neurodegenerative disease.

### 3.4 Machine learning biomarker selection

**FIGURE 2:**
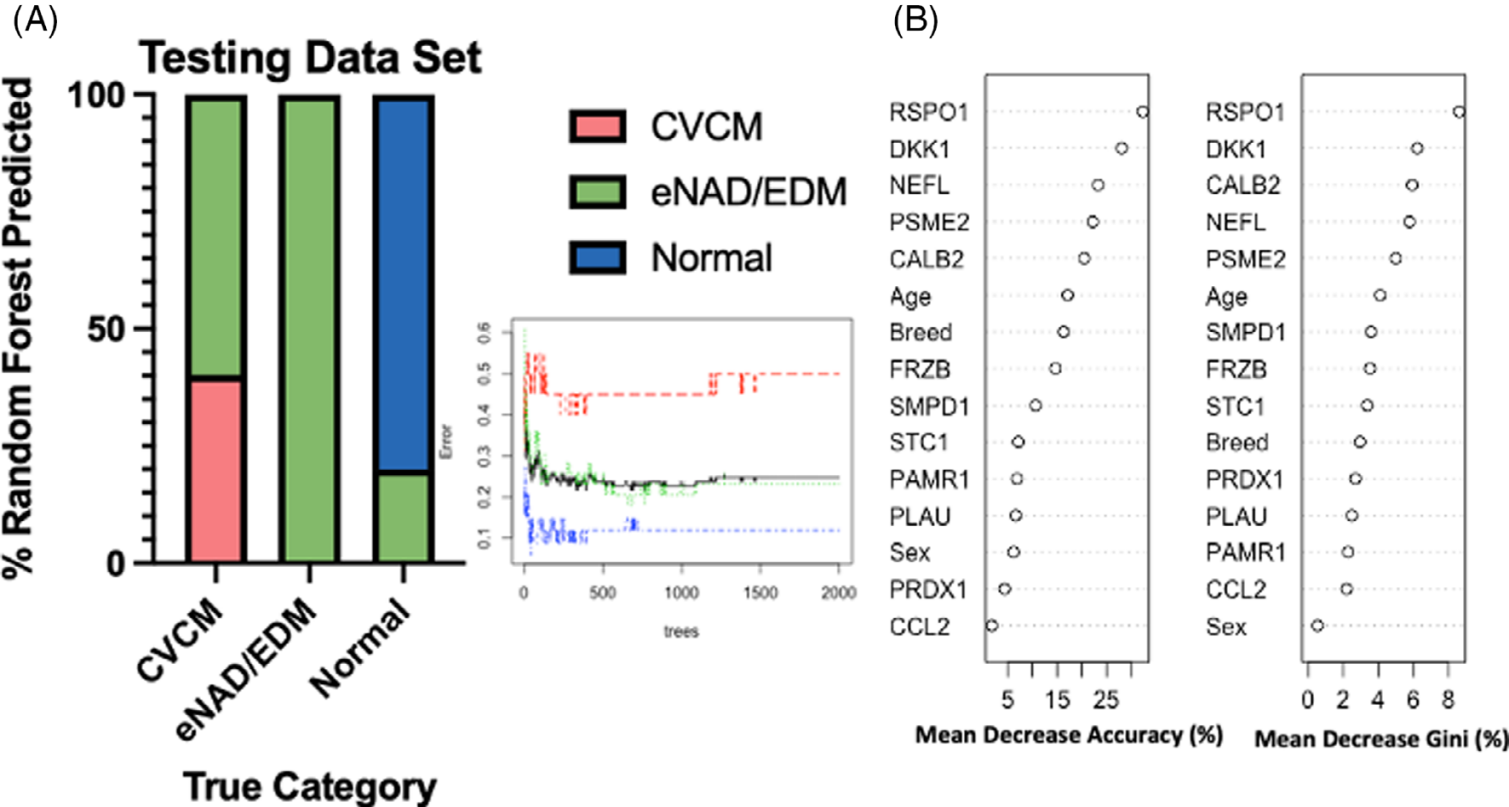
Random forest analysis of CSF. Testing data set (A) shows classification of the three groups as percentage of correctly assigned animals. Inset in (A) error plot of the training data set for CVCM (red), eNAD/EDM (green), normal (blue), and overall (black). The x‐axis indicates the error rate for classification and the y‐axis the number of trees used in the classification. Accuracy in the testing set was highest for eNAD/EDM (100%), followed by normal (80%) and CVCM (40%). Importantly, no normal horses were misclassified in the ataxia groups. Mean decrease in accuracy and Gini plots (B) ranks the proteins and categorical factors (age, breed, and sex) used in decisions by the percentage decrease in accuracy or Gini if that feature were to be removed from the model.

Random forest classifier models for serum, CSF and a merged CSF/serum dataset were constructed. Two‐thousand and one trees were used for each sample matrix. In all 3 datasets, error values leveled at approximately 500 trees for the training set (Figure 2, Supplementary Figure 2). Testing sets using CSF and merged datasets had the highest accuracy for correctly classifying animals by group. Both datasets performed best for normal and eNAD/EDM horses, correctly classifying them with >80% accuracy for each (Figure 2, Supplementary Figure 3). Mean decrease accuracy plots indicated a group of proteins that were driving classification in the random forest model.

**FIGURE 3:**
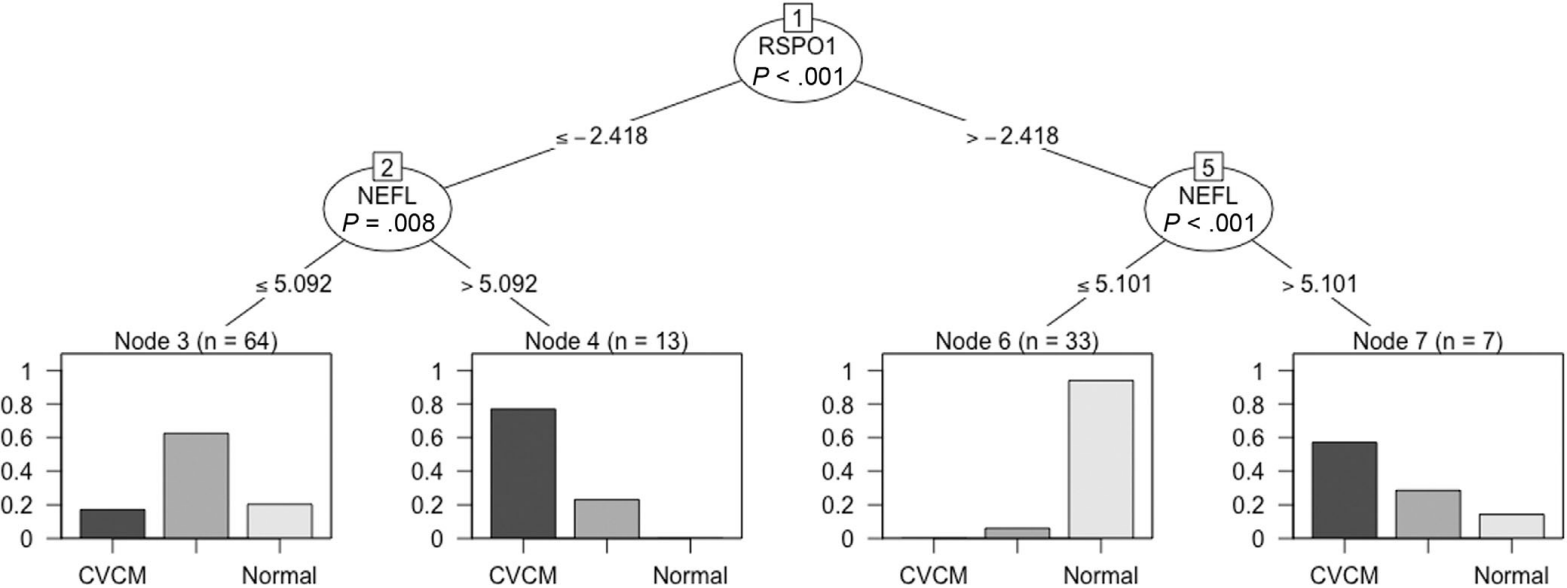
Conditional inference tree for CSF. A 2‐protein model with RSPO1 and NEFL had the highest accuracy for prediction of normal horses (87.2%), CVCM (84.6%) and eNAD/EDM (73.5%). NEFL, neurofilament‐light; RSPO1, R‐Spondin 1.

**FIGURE 4:**
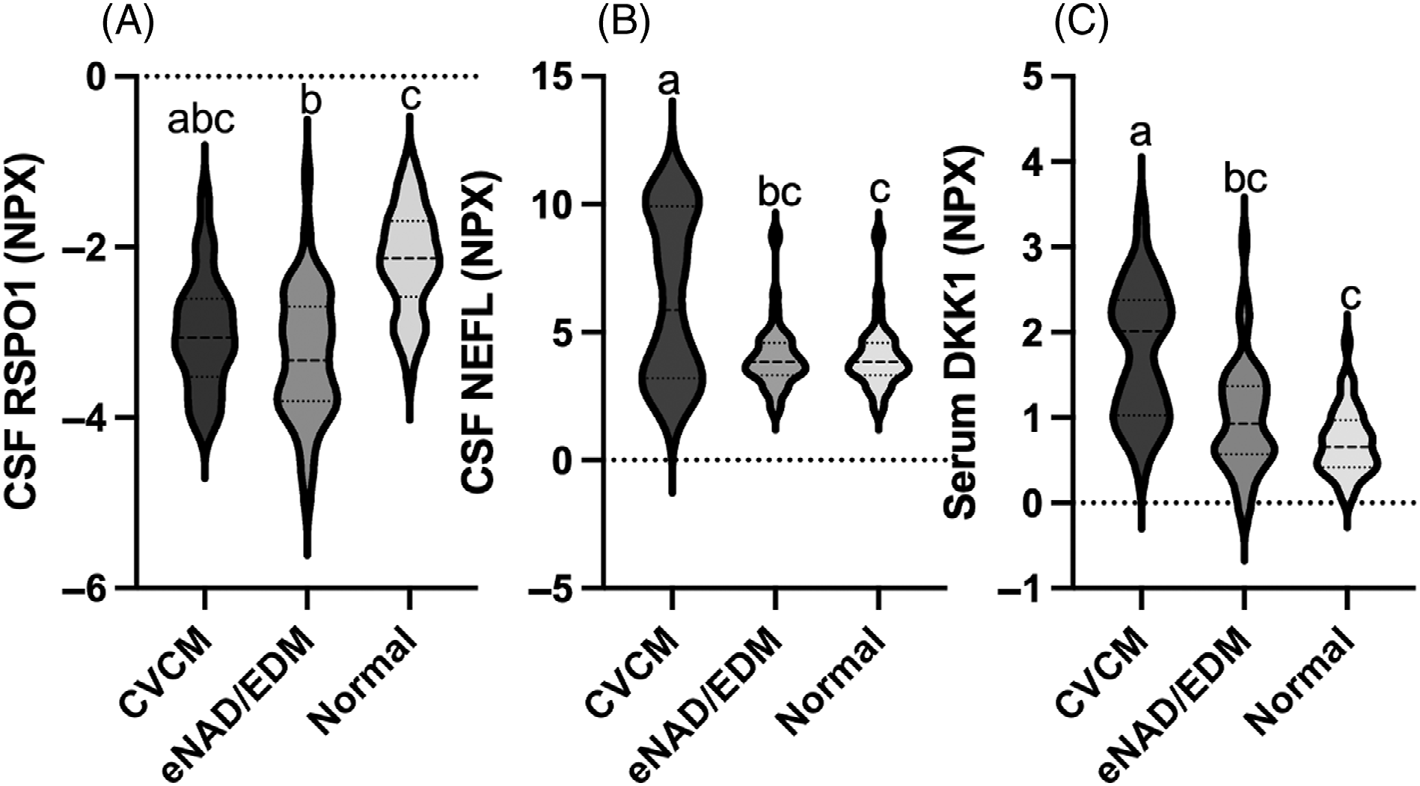
Violin plots of potential biomarkers as defined by random forest and conditional inference models in CSF and merged data sets; CSF RSPO1 (A), CSF NEFL (B) and serum DKK1 (C). Different letters indicate significant difference between groups based on pair‐wise post hoc ANCOVA with correction for multiple testing (*P* < .05). DKK1, Dickkoph 1; NEFL, neurofilament‐light; RSPO1, R‐Spondin 1.

Conditional inference model selection of biomarkers mirrored the top‐ranking proteins as determined by random forest. In serum, 2 proteins were selected: WAP, follistatin/kazal, immunoglobulin, kunitz and neutrin domain containing 1 (WFKKN1) and Dickkoph‐1 (DKK1). In serum, this modeling offered diagnostic accuracies of 67.5% for eNAD/EDM, 87.2% for CVCM and 76.9% for normal horses (Supplementary Figure 4). In CSF, a 2‐protein test with R‐spondin‐1 (RSPO1; Figure 4A) and neurofilament‐light (NEFL; Figure 4B) was selected (Figure 3). Profiling these 2 CSF proteins resulted in a prediction accuracy of 73.5% for eNAD/EDM, 84.6% for CVCM and 87.2% for normal horses. For the merged data set, 2 CSF proteins (RSPO1 and NEFL) and 1 serum protein (DKK1; Figure 4C) were selected, with prediction accuracies of 72.7% for eNAD/EDM, 84.5% for CVCM and 85.5% for normal horses (Supplementary Figure 5).

## 4 DISCUSSION

We demonstrated the effective use of PEA technology for the first time using equine serum and CSF sample matrices. Use of this platform allowed for the identification of previously unidentified proteins in equine CSF and serum. Importantly, with the aid of machine learning algorithms, we found that a limited set of novel CSF proteins can accurately discriminate between not only neurologically normal and abnormal horses, but also by neurodegenerative etiology. These findings add to the catalogue of proteins detectable in equine CSF and serum, while also proposing biomarker candidates that will enhance diagnostic resolution for equine spinal ataxia.

The equine CSF proteome is a rich, but relatively untapped source of protein biomarkers. Previous work identified 320 proteins in CSF from 6 reportedly healthy horses using a 2‐dimensional liquid chromatography tandem mass spectroscopy (2D‐LC‐MS/MS) approach.^11^ None of the proteins identified in our study were identified in the previous study. Although both techniques are highly sensitive, LC‐MS/MS is dependent on a high‐quality annotation by which to assign protein identity. Given the limitations of the equine proteome annotation, highly abundant proteins are often well characterized, whereas rare proteins may not be identified, even if present.^12^ Proximity extension assay technology as a targeted sequencing‐based technology is not reliant on protein annotation, nor affected by the presence of highly abundant proteins such as albumin and globulins.^10^ As such, it is well suited to detect and qualitatively define rare proteins within a sample matrix. The lack of concordance among studies is likely caused by the inherent difference between spectroscopy‐based proteomics and the sequencing‐based approach employed in our study. Additionally, the former study only included healthy horses and therefore the repertoire of identifiable proteins may have been narrowed by lack of an active pathologic process. Although PEA technology allows for identification of proteins with high fidelity, in its current form, it is still a targeted technology and only detects proteins for which the assay has probes. Therefore, the absence of proteins in 1 study compared to another is an expected finding. Collectively, both approaches add to the catalogue of known proteins present in equine CSF.

Although the PEA assay has probes for 368 proteins, neither CSF nor serum approached the total number of possible detectable proteins. This result, in part, may be attributed to the cross‐species platform. All probes in this platform were developed for human‐derived proteins. Pilot data generated from our laboratory indicated that highly conserved proteins (>80% protein‐protein identity) performed well using this platform. The technology has been used previously for equine synovial samples, where it was able to accurately discriminate among various cyto‐ and chemokines.^13^ Given the dual‐antibody design, the risk of false positive detection of proteins is negligible. When used in nonhuman species, the technology is likely to underrepresent the number of detectable proteins in a sample. Our findings support this hypothesis. Additionally, the sample matrix is an important contributor to the proteins available for detection. The discrepancy between CSF and serum was an expected finding, similar to previous work published with human cohorts.^14^, ^15^ It is intuitive that a sample matrix with lower amounts of protein (e.g., CSF) will have a decreased complexity of proteins present.

Machine learning models offer an unbiased and tractable technique to distill high‐dimensional data into useful diagnostic sets. Random forest and conditional inference, although similar, have some fundamental differences. Namely, random forest takes an ensemble approach, with predictions averaged among the sample parameters.^16^ Conditional inference selects the feature or features that best distinguishes among groups in a more purely binary fashion. As such, random forest enables the effect of a feature on prediction to be viewed relative to each other feature (ie, size of effect of 1 protein compared to another), whereas conditional inference defines each feature by binary partition. In our study, random forest helped establish the rank importance of each relevant protein in classification of disease phenotype, whereas conditional inference allowed for the discovery of potential diagnostic protein sets. Importantly, the conditional inference sets are informed by the smallest number of proteins to achieve the most accurate classification based on a statistically significant difference. This approach has practical significance for future development of these biomarkers as diagnostic tests, with fewer markers aiding clinical interpretation.^17^ Use of the 2 models resulted in similar classification accuracies for both eNAD/EDM and normal horses, whereas conditional inference was far more accurate for CVCM. This result is a consequence of the smaller sample size of the CVCM group and the requirement to train the random forest model, further decreasing the sample size. By comparison, conditional inference does not introduce a training data set and the sample size is optimized, and this difference accounts for the improved performance of the CVCM dataset. Performance of these models suggests that addition of these biomarkers to existing diagnostic modalities will aid in the accurate diagnosis of spinal ataxias resulting from eNAD/EDM or CVCM. Although findings from our are encouraging, further work to validate these biomarkers in a replication study will be required before using these biomarkers in clinical practice.

Neurofilament light (NEFL) was identified as an important protein for group classification in our study, with relative amounts in CSF and serum highest for CVCM. Neurofilament light is part of the neurofilament family of proteins important for the cytoskeletal structure of the axon.^6^ Increases in this protein, both in serum and CSF, have been well characterized in neurodegenerative diseases of humans including Alzheimer's disease, frontotemporal dementia (FTD) and amyotrophic lateral sclerosis (ALS).^6^ Similarly, concentrations increase with acute trauma, including traumatic brain injury (TBI) and spinal cord injury (SCI).^18^, ^19^, ^20^ Neurofilament light has not been evaluated previously in horses. Phosphorylated neurofilament heavy (pNfH), a polypeptide from the same family, is the best characterized neurofilament in horses with neurologic disease.^7^, ^21^, ^22^, ^23^, ^24^, ^25^ Previous studies found increases of pNfH in CSF of horses both with CVCM and eNAD/EDM.^7^ As such, increases were consistent with neurodegenerative disease, but did not differentiate etiology. In our study, NEFL differentiated CVCM from both eNAD/EDM and normal horses. However, before use of NEFL as a diagnostic marker, further work is required to evaluate the protein quantitatively. Ultrasensitive NEFL quantification, such as single molecule analysis (SIMOA), has not yet been reported in the horse. In dogs, use of this technology allowed for the quantification of NEFL in serum or plasma and CSF at picogram concentrations and showed promise as a diagnostic, prognostic, and treatment response biomarker.^26^, ^27^, ^28^, ^29^

In addition to NEFL, other proteins that are under evaluation as biomarkers in neurologic diseases of humans were detected. These included Dickkoph‐1 (DDK1), an important inhibitor of WNT signaling.^30^ Increases of DKK1 have been identified in SCI and may be related to severity of injury.^31^ Additionally, DKK1 is under investigation as a target for intervention in Alzheimer's disease.^32^ R‐spondin 1 (RSPO1) was the highest ranked marker following random forest analysis and conditional inference modeling in CSF. R‐spondin 1 is a positive regulator of canonical WNT/β‐catenin signaling and has been shown to increase in presymptomatic and affected human patients with familial Alzheimer's disease.^33^, ^34^ Interestingly, in our study, it was decreased in both CVCM and eNAD/EDM compared to normal horses. The RSPO1 increase in humans is considered to be a consequence of the pathology associated with Alzheimer's disease, because spondins have shown protective potential in murine Alzheimer's models.^35^ Therefore, despite the difference in trajectory, enrichment in normal horses may indicate a protective function. Serum WAP, follistatin/kazal, immunoglobulin, kunitz, and neutrin domain containing 1 (WFIKN1) were important predictors in the serum only modeling. In CSF, decreased abundance of this protein has been associated with treatment for central nervous system leukemia after acute lymphocytic leukemia.^36^ Using the same Olink panel, increased plasma WFIKN1 was strongly associated with genetic predisposition to schizophrenia.^37^ The role of WFIKN1 in neurologic disease is yet to be fully defined, but it is a known regulator of TGFβ and this signaling pathway is intimately associated with neuronal maintenance, function, and degeneration.^38^ Collectively, the consistency of protein signals between our study and those performed in humans and murine models indicates the conservation of molecular mechanisms underpinning neurodegeneration. This consistency gives additional confidence in the use of PEA technology for evaluation of samples from horses, with similar signals detected when compared to human and murine data.

Additional potential biomarkers of interest were detected our study, including CSF calretinin (CALB2) and serum superoxide dismutase‐2 (SOD2). Calretinin previously has been used to define the tracts that undergo degeneration in eNAD/EDM.^39^ However, detection of this protein in CSF as a marker of neurodegeneration has not been demonstrated previously and represents a novel finding. Increased in SOD2 helped distinguish the CVCM horse serum proteome from the eNAD/EDM horse proteome. Superoxide dismutase‐2 is the mitochondrial member of the SOD family and, similar to our findings, it is decreased in murine models of SCI.^40^ These findings again validate the use of Olink technology for biomarker discovery in nonhuman species, including horses, as it reflects known molecular components of neurodegeneration in that species.

Our findings highlight several potential biomarkers for neurodegenerative disease in horses. However, our study had some limitations. First, unequal distribution of disease groups may have affected our machine learning algorithms, particularly with the smaller CVCM sample set. Because of the strict disease category approach that was taken (ie, necropsy was required), the available pool of samples was decreased. Despite the smaller size, the effect magnitude was higher and somewhat more homogeneous compared to both the normal and eNAD/EDM groups, allowing for partial abrogation of the sample size deficit. The second limitation is that our study does not include infectious, inflammatory, or traumatic causes of spinal ataxia. Validation of these potential biomarkers will require evaluation of these additional causes of spinal ataxia to ensure the diagnostic specificity for the intended neurodegenerative disease. However, diagnostic tests currently exist for many of the infectious causes of spinal ataxia in horses. The final limitation is that our study represents only a snapshot in time for both disease groups. Temporal dynamics of these biomarkers may play a role in their diagnostic value, and therefore prospective repeated sampling studies evaluating these markers will be valuable to increase confidence in our current findings.

In conclusion, ours is the first study to report on the use of PEA proteomic technology for equine serum and CSF samples. This technology, in tandem with machine learning algorithms, defines highly accurate dual‐ and triplex biomarkers for diagnosis of CVCM and eNAD/EDM in horses. Future studies to validate these markers by development of quantitative assay panels in larger replication cohorts are required before clinical adoption of these biomarkers.

## CONFLICT OF INTEREST DECLARATION

Authors declare no conflict of interest.

## OFF‐LABEL ANTIMICROBIAL DECLARATION

Authors declare no off‐label use of antimicrobials.

## INSTITUTIONAL ANIMAL CARE AND USE COMMITTEE (IACUC) OR OTHER APPROVAL DECLARATION

Authors declare no IACUC or other approval was needed.

## HUMAN ETHICS APPROVAL DECLARATION

Authors declare human ethics approval was not needed for this study.

## Supporting information


**Data S1:** Supporting Information
