# Supplementary material for: Cerebrospinal fluid and serum proteomic profiles accurately distinguish neuroaxonal dystrophy from cervical vertebral compressive myelopathy in horses
Source: J Vet Intern Med. 2023 Mar 16;37(2):689–96. doi: 10.1111/jvim.16660 (PMC10061172; doi:10.1111/jvim.16660)
Supplement: Supplementary file 1 — Data S1: Supporting Information [file JVIM-37-689-s001.pdf]

Supplementary Table 1. Proteins identified in ≥ 50% of samples analyzed using the Target 96 Neuro-Exploratory panel for both serum and CSF with eNAD/EDM (*n=5)* and age/sex matched controls (*n=5)* to pilot the technology using equine derived samples. *denotes proteins that were detected in the subsequent study using Olink Explorer 387.			
**Assay**	**OlinkID**	**UniProt**	**Sample Matrix**
CRADD	OID05129	P78560	Serum Only
CETN2	OID05133	P41208	Serum Only
SMOC1	OID05135	Q9H4F8	Serum and CSF
ADGRB3	OID05521	O60242	Serum and CSF
KLB	OID05137	Q86Z14	Serum Only
CDH17	OID05138	Q12864	Serum Only
GPNMB	OID05139	Q14956	Serum Only
ATP6V1F	OID05143	Q16864	Serum Only
ANXA10	OID05147	Q9UJ72	Serum Only
RPS6KB1	OID05150	P23443	Serum Only
CRIP2	OID05154	P52943	Serum and CSF
ADAM15	OID05162	Q13444	Serum and CSF
FGFR2	OID05166	P21802	Serum and CSF
NAA10	OID05168	P41227	Serum Only
SFRP1	OID05171	Q8N474	Serum and CSF*
PRTFDC1	OID05176	Q9NRG1	Serum Only
TBCB	OID05181	Q99426	Serum Only*
NPM1	OID05182	P06748	Serum and CSF
ASGR1	OID05183	P07306	Serum and CSF
COL4A3BP	OID05185	Q9Y5P4	Serum Only
PSG1	OID05186	P11464	Serum Only
PSME1	OID05187	Q06323	Serum Only*
PTS	OID05193	Q03393	Serum Only
FUT8	OID05195	Q9BYC5	Serum and CSF

TPPP3	OID05196	Q9BW30	Serum Only
PFDN2	OID05197	Q9UHV9	Serum Only
AARSD1	OID05202	Q9BTE6	Serum Only
PHOSPHO1	OID05203	Q8TCT1	Serum Only
NEFL	OID05206	P07196	Serum and CSF*
HMOX2	OID05207	P30519	Serum Only
KIF1BP	OID05213	Q96EK5	Serum Only
PPP3R1	OID05214	OID05214	Serum and CSF*
ILKAP	OID05215	Q9H0C8	Serum Only
PMVK	OID05218	Q15126	Serum Only*
WWP2	OID05219	O00308	Serum and CSF*
GGT5	OID05221	P36269	Serum Only*
CLSTN1	OID05158	O94985	CSF Only
CD302	OID05152	Q8IX05	CSF Only
NXPH1	OID05167	P58417	CSF Only*
MAD1L1	OID05191	Q9Y6D9	CSF Only
ISLR2	OID05216	Q6UXK2	CSF Only

**Supplementary Table 2. Post-hoc ANCOVA analysis of serum proteins present in ≥ 50% of samples using the Olink Explorer 387 platform that were significantly differently abundant following correction for multiple comparisons. Proteins are listed in order of significance. A Bonferroni adjusted P-value <0.05 was considered significant.**							
**Assay**	**OlinkID**	**UniProt**	**contrast**	**estimate**	**conf.low**	**conf.high**	**Adjusted_pval**
CD164	OID21080	Q04900	NAD_EDM - Normal	0.325567	0.161252	0.489883	2.21E-05
NEFL	OID20871	P07196	CVCM - Normal	1.130536	0.507985	1.753088	1.04E-04
RHOC	OID20950	P08134	CVCM - NAD_EDM	-0.87318	-1.43319	-0.31317	9.69E-04
TARBP2	OID20870	Q15633	CVCM - NAD_EDM	-0.78036	-1.28217	-0.27854	1.00E-03
BIN2	OID21067	Q9UBW5	NAD_EDM - Normal	0.274369	0.094348	0.454391	1.29E-03
THY1	OID21050	P04216	NAD_EDM - Normal	0.285633	0.096942	0.474323	1.40E-03
TBC1D17	OID20844	Q9HA65	CVCM - NAD_EDM	-0.7769	-1.29659	-0.25722	1.63E-03
DKK1	OID21066	O94907	CVCM - NAD_EDM	0.51436	0.168298	0.860421	1.75E-03
CALCA	OID20983	P01258	CVCM - Normal	-0.88985	-1.51492	-0.26479	2.85E-03
PBLD	OID20839	P30039	CVCM - Normal	-0.53554	-0.91672	-0.15435	3.29E-03
DNMBP	OID20956	Q6XZF7	CVCM - NAD_EDM	-0.92552	-1.58511	-0.26593	0.003333
MESD	OID21099	Q14696	CVCM - NAD_EDM	-0.26999	-0.46625	-0.07373	0.004102
TBCC	OID20942	Q15814	CVCM - Normal	-0.56146	-0.97206	-0.15086	0.004363
MITD1	OID20959	Q8WV92	CVCM - NAD_EDM	-0.77114	-1.34233	-0.19996	0.004962
GGT5	OID20909	P36269	CVCM - NAD_EDM	0.646726	0.166617	1.126835	0.005074
THY1	OID21050	P04216	CVCM - Normal	0.340517	0.087234	0.5938	0.005173
C19orf12	OID20804	Q9NSK7	NAD_EDM - Normal	-0.43021	-0.75083	-0.10958	0.005274
PPP3R1	OID20902	P63098	CVCM - NAD_EDM	-0.64736	-1.13947	-0.15526	0.006388
TBCB	OID20993	Q99426	NAD_EDM - Normal	-0.49305	-0.87101	-0.11509	0.006915
DKK1	OID21066	O94907	CVCM - Normal	0.466918	0.104408	0.829428	0.007781
SFRP1	OID20984	Q8N474	CVCM - Normal	1.399865	0.312303	2.487426	0.007828
TST	OID20868	Q16762	CVCM - Normal	-0.70081	-1.24563	-0.15599	0.007876

APRT	OID20927	P07741	CVCM - NAD_EDM	-1.24049	-2.2075	-0.27349	0.008073
GGT5	OID20909	P36269	CVCM - Normal	0.631417	0.128488	1.134345	0.009785
LBR	OID21034	Q14739	NAD_EDM - Normal	0.735311	0.147206	1.323416	0.010144
ALDH1A1	OID21128	P00352	CVCM - Normal	-0.34266	-0.6175	-0.06783	0.010394
NEFL	OID20871	P07196	CVCM - NAD_EDM	0.738595	0.144291	1.3329	0.010687
PTEN	OID20794	P60484	CVCM - NAD_EDM	-0.29912	-0.53998	-0.05827	0.010752
TBCC	OID20942	Q15814	NAD_EDM - Normal	-0.37666	-0.68255	-0.07077	0.011563
TST	OID20868	Q16762	NAD_EDM - Normal	-0.49787	-0.90375	-0.09199	0.011942
C19orf12	OID20804	Q9NSK7	CVCM - Normal	-0.52162	-0.952	-0.09124	0.013195
BAX	OID20856	Q07812	CVCM - NAD_EDM	-0.99399	-1.82104	-0.16694	0.014132
CHMP1A	OID20930	Q9HD42	CVCM - Normal	-0.60938	-1.11805	-0.10072	0.014502
WFIKKN1	OID20939	Q96NZ8	CVCM - Normal	0.455095	0.072153	0.838037	0.015463
MESD	OID21099	Q14696	CVCM - Normal	-0.24383	-0.44942	-0.03825	0.01571
DNMBP	OID20956	Q6XZF7	NAD_EDM - Normal	0.603628	0.088889	1.118366	0.017164
SUSD2	OID21098	Q9UGT4	NAD_EDM - Normal	-0.31126	-0.57764	-0.04489	0.017643
NXPH1	OID20849	P58417	NAD_EDM - Normal	-0.57665	-1.07199	-0.08131	0.018154
BST2	OID21029	Q10589	NAD_EDM - Normal	-0.34222	-0.63817	-0.04627	0.019107
JAM2	OID21064	P57087	CVCM - Normal	0.320304	0.032203	0.608405	0.025436
ADAM22	OID21001	Q9P0K1	CVCM - NAD_EDM	-0.58518	-1.11921	-0.05115	0.028121
APRT	OID20927	P07741	NAD_EDM - Normal	0.818964	0.064323	1.573605	0.030027
MASP1	OID20954	P48740	CVCM - NAD_EDM	-0.27784	-0.53443	-0.02125	0.03048
DUSP3	OID20827	P51452	CVCM - NAD_EDM	0.743752	0.055135	1.432369	0.031002
GLB1	OID20949	P16278	NAD_EDM - Normal	0.717589	0.052848	1.38233	0.03111
PMVK	OID20850	Q15126	CVCM - NAD_EDM	-0.8291	-1.60331	-0.05489	0.032796
PARK7	OID21160	Q99497	NAD_EDM - Normal	0.412715	0.025238	0.800192	0.033972
TFF1	OID21154	P04155	CVCM - NAD_EDM	-0.13723	-0.26736	-0.00711	0.03621
PECAM1	OID21131	P16284	CVCM - NAD_EDM	-0.18414	-0.35929	-0.00898	0.03695
NOS3	OID20834	P29474	CVCM - Normal	0.522493	0.021814	1.023172	0.038699
PSME2	OID20989	Q9UL46	CVCM - Normal	-0.3537	-0.69277	-0.01463	0.038793
NSFL1C	OID21022	Q9UNZ2	NAD_EDM - Normal	0.716216	0.02814	1.404293	0.039322
SCARB1	OID20805	Q8WTV0	NAD_EDM - Normal	-0.45798	-0.90083	-0.01514	0.040925

FMNL1	OID20884	O95466	CVCM - Normal	-0.73643	-1.45242	-0.02044	0.042316
GP6	OID21091	Q9HCN6	NAD_EDM - Normal	0.216845	0.004951	0.42874	0.043631
PAMR1	OID21153	Q6UXH9	CVCM - Normal	0.241835	0.005208	0.478461	0.04398
ITGAM	OID21071	P11215	NAD_EDM - Normal	0.124289	0.002142	0.246437	0.045152
HARS	OID21086	P12081	CVCM - NAD_EDM	-0.39745	-0.78853	-0.00637	0.045485
CXCL8	OID20997	P10145	NAD_EDM - Normal	0.531976	0.007266	1.056686	0.046137
MITD1	OID20959	Q8WV92	NAD_EDM - Normal	0.450839	0.005093	0.896584	0.046793
SERPINB9	OID20932	P50453	CVCM - Normal	-0.62873	-1.25251	-0.00494	0.047757
SOD2	OID21114	P04179	CVCM - NAD_EDM	-0.43211	-0.86292	-0.0013	0.049133

Supplementary Table 3. Post-hoc ANCOVA analysis of CSF proteins present in ≥50% of samples using the Olink Explorer 387 platform that were significantly differently abundant following correction for multiple comparisons. Proteins are listed in order of significance. Adjusted P-value <0.05 considered significant.							
**Assay**	**OlinkID**	**UniProt**	**contrast**	**estimate**	**conf.low**	**conf.high**	**Adjusted_pval**
CALB2	OID20801	P22676	CVCM - Normal	0.939744	4.73E-01	1.406741	1.64E-05
RSPO1	OID20938	Q2MKA7	NAD_EDM - Normal	-0.77522	-1.16E+00	-0.38597	2.00E-05
NEFL	OID20871	P07196	CVCM - Normal	2.479153	1.21E+00	3.746837	2.83E-05
SULT1A1	OID21031	P50225	NAD_EDM - Normal	-0.70169	-1.11E+00	-0.2969	2.19E-04
DKK1	OID21066	O94907	CVCM - Normal	0.868977	3.51E-01	1.386758	3.55E-04
PSME2	OID20989	Q9UL46	NAD_EDM - Normal	-0.6063	-9.93E-01	-0.21981	8.96E-04
PSME1	OID20969	Q06323	CVCM - Normal	-0.73693	-1.23E+00	-0.23951	1.81E-03
SIGLEC5	OID21082	O15389	CVCM - NAD_EDM	-0.4153	-7.00E-01	-0.13052	2.18E-03
PSME1	OID20969	Q06323	NAD_EDM - Normal	-0.53154	-9.02E-01	-0.16098	2.62E-03
PSME2	OID20989	Q9UL46	CVCM - Normal	-0.74137	-1.26E+00	-0.22258	2.73E-03
APRT	OID20927	P07741	NAD_EDM - Normal	-0.47945	-8.34E-01	-0.12458	4.93E-03
SULT1A1	OID21031	P50225	CVCM - Normal	-0.72614	-1.27E+00	-0.18278	5.49E-03
SNCG	OID20971	O76070	NAD_EDM - Normal	0.251777	6.28E-02	0.440786	5.66E-03
WWP2	OID20926	O00308	CVCM - Normal	-0.6377	-1.13E+00	-0.14749	7.10E-03
FABP5	OID21043	Q01469	CVCM - NAD_EDM	0.951041	2.19E-01	1.682717	7.15E-03
NAAA	OID20931	Q02083	CVCM - Normal	0.34098	7.57E-02	0.606293	7.94E-03
SUSD2	OID21098	Q9UGT4	CVCM - Normal	0.452657	1.00E-01	0.804945	7.95E-03
NEFL	OID20871	P07196	CVCM - NAD_EDM	1.554647	3.44E-01	2.764812	7.97E-03
FABP5	OID21043	Q01469	CVCM - Normal	0.965136	1.99E-01	1.731588	9.53E-03
PAMR1	OID21153	Q6UXH9	CVCM - Normal	0.712564	1.40E-01	1.28531	1.06E-02
TXLNA	OID20952	P40222	NAD_EDM - Normal	-0.30524	-5.53E-01	-0.05747	1.15E-02

SFRP1	OID20984	Q8N474	NAD_EDM - Normal	-0.79839	-1.45E+00	-0.15007	1.16E-02
CCL2	OID21004	P13500	CVCM - Normal	1.129113	2.12E-01	2.046612	1.16E-02
CALB2	OID20801	P22676	CVCM - NAD_EDM	0.544303	9.85E-02	0.990111	1.24E-02
GKN1	OID20885	Q9NS71	CVCM - Normal	0.51649	8.94E-02	0.943595	1.34E-02
PILRA	OID21129	Q9UKJ1	CVCM - NAD_EDM	0.447317	7.65E-02	0.818115	1.37E-02
FOSB	OID20791	P53539	CVCM - Normal	0.274892	4.40E-02	0.505782	1.52E-02
EZR	OID21017	P15311	CVCM - NAD_EDM	0.213294	3.10E-02	0.395552	1.74E-02
TXLNA	OID20952	P40222	CVCM - Normal	-0.38906	-7.22E-01	-0.05647	1.75E-02
SIRT5	OID20820	Q9NXA8	CVCM - NAD_EDM	0.263506	3.49E-02	0.492142	1.96E-02
MASP1	OID20954	P48740	NAD_EDM - Normal	0.133277	1.76E-02	0.248918	1.96E-02
CALB2	OID20801	P22676	NAD_EDM - Normal	0.395441	4.75E-02	0.743344	2.17E-02
DKK1	OID21066	O94907	CVCM - NAD_EDM	0.5617	6.74E-02	1.055988	2.17E-02
THY1	OID21050	P04216	CVCM - Normal	-0.06298	-1.21E-01	-0.00518	2.92E-02
HARS	OID21086	P12081	NAD_EDM - Normal	-0.31979	-6.18E-01	-0.02184	3.23E-02
NOMO1	OID21106	Q15155	CVCM - Normal	0.286211	1.82E-02	0.554178	3.34E-02
IGF2R	OID21146	P11717	NAD_EDM - Normal	-0.22499	-4.36E-01	-0.01348	3.43E-02
PLAU	OID21124	P00749	NAD_EDM - Normal	0.457392	2.36E-02	0.891221	3.63E-02
EZR	OID21017	P15311	CVCM - Normal	0.201099	1.02E-02	0.392021	3.65E-02
FRZB	OID20968	Q92765	CVCM - NAD_EDM	0.745323	3.38E-02	1.456817	3.78E-02
ANXA3	OID21036	P12429	CVCM - Normal	0.176811	7.79E-03	0.345831	3.81E-02
SMPD1	OID21028	P17405	CVCM - NAD_EDM	-0.31368	-6.14E-01	-0.0132	3.86E-02
APRT	OID20927	P07741	CVCM - Normal	-0.49311	-9.69E-01	-0.01676	4.07E-02
PAMR1	OID21153	Q6UXH9	NAD_EDM - Normal	0.439905	1.32E-02	0.866589	4.17E-02
TBCC	OID20942	Q15814	NAD_EDM - Normal	-0.26414	-5.22E-01	-0.0067	4.29E-02
CX3CL1	OID20976	P78423	NAD_EDM - Normal	-0.34337	-6.78E-01	-0.00852	4.31E-02
LPO	OID20963	P22079	CVCM - Normal	0.676457	1.65E-02	1.33644	4.32E-02
PDCD5	OID21021	O14737	CVCM - NAD_EDM	0.424762	5.49E-03	0.844035	4.63E-02
OBP2B	OID20980	Q9NPH6	CVCM - NAD_EDM	0.502511	3.86E-03	1.001167	4.78E-02
THBS2	OID21104	P35442	CVCM - Normal	0.444463	2.85E-03	0.886074	4.82E-02

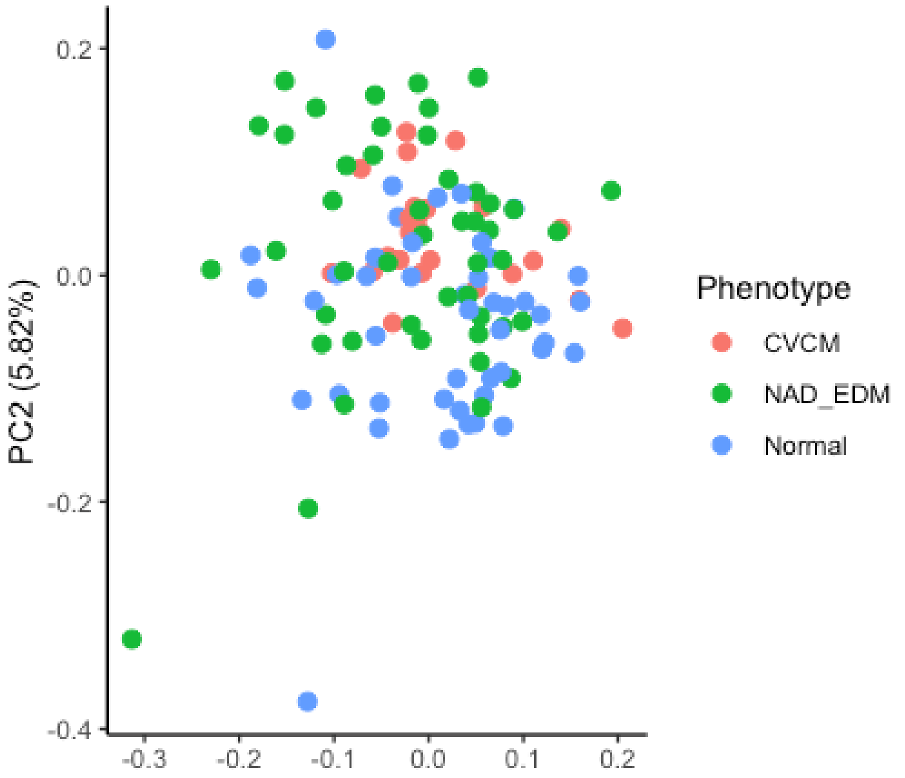

Supplementary Figure 1. Principal component plot serum proteins showing the first two components (PC1 and PC2). Using proteins with a missingness ≤50% values were converted to eigenvectors and plotted. Each dot represents an individual horse from the CVCM group (n=25; salmon), eNAD/EDM (n=47; blue) or normal (n=45; green).

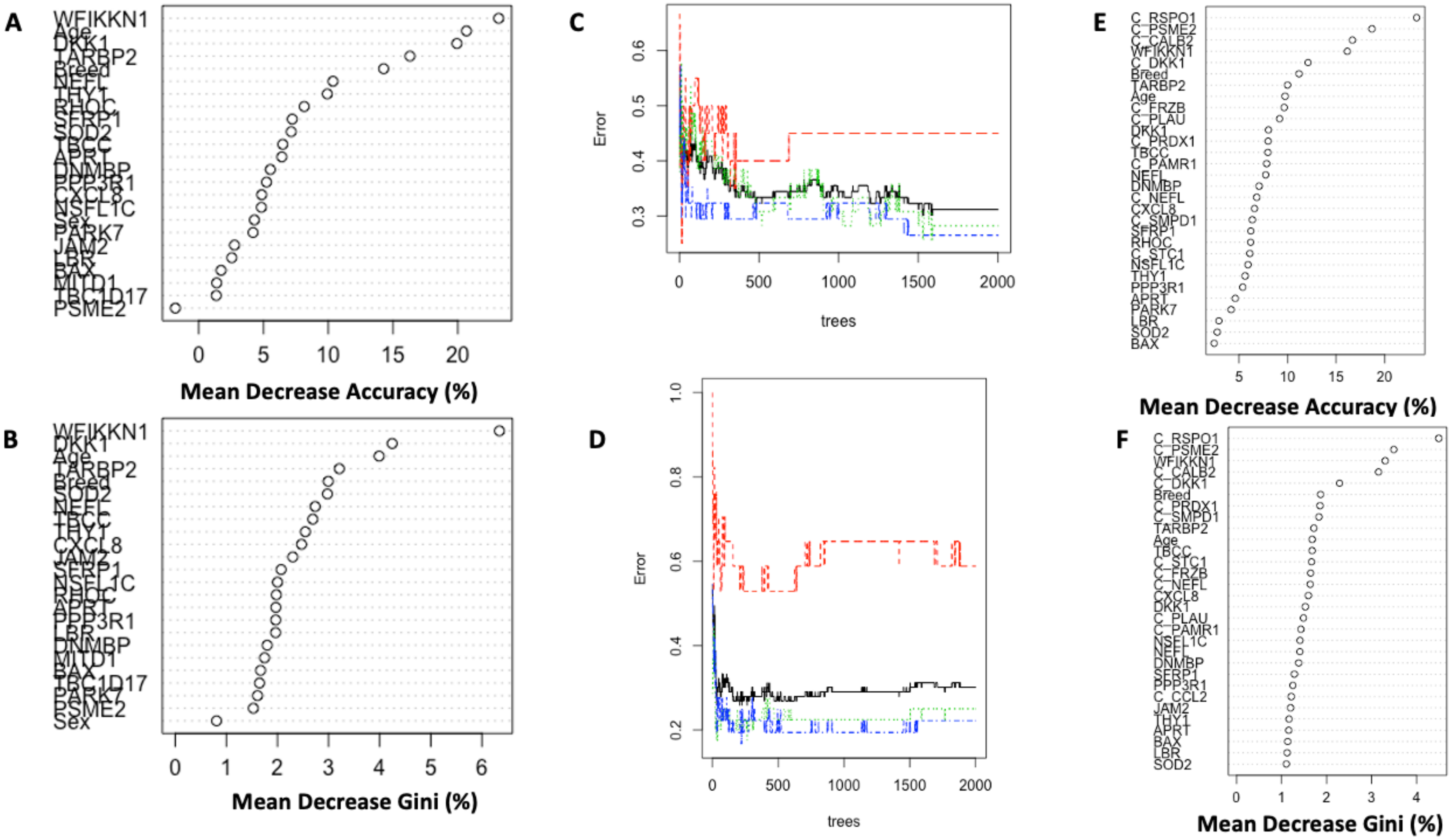

Supplementary Figure 2. Random Forest Analysis of serum (A,B,C) and merged serum/CSF data (D,E,F). Error plot (C,D) of the training data set for CVCM (red), eNAD/EDM (green), normal (blue) and overall (black). The x-axis indicates the error rate for classification and the y-axis the number of trees used in the classification. Mean decrease in Accuracy (A,B) and mean decrease Gini ranks the proteins and categorical factors (age, breed and sex) used in decision by the percentage decrease in accuracy if that feature were to be removed from the model. Proteins with the prefix C_ denotes CSF proteins in the merged data set.

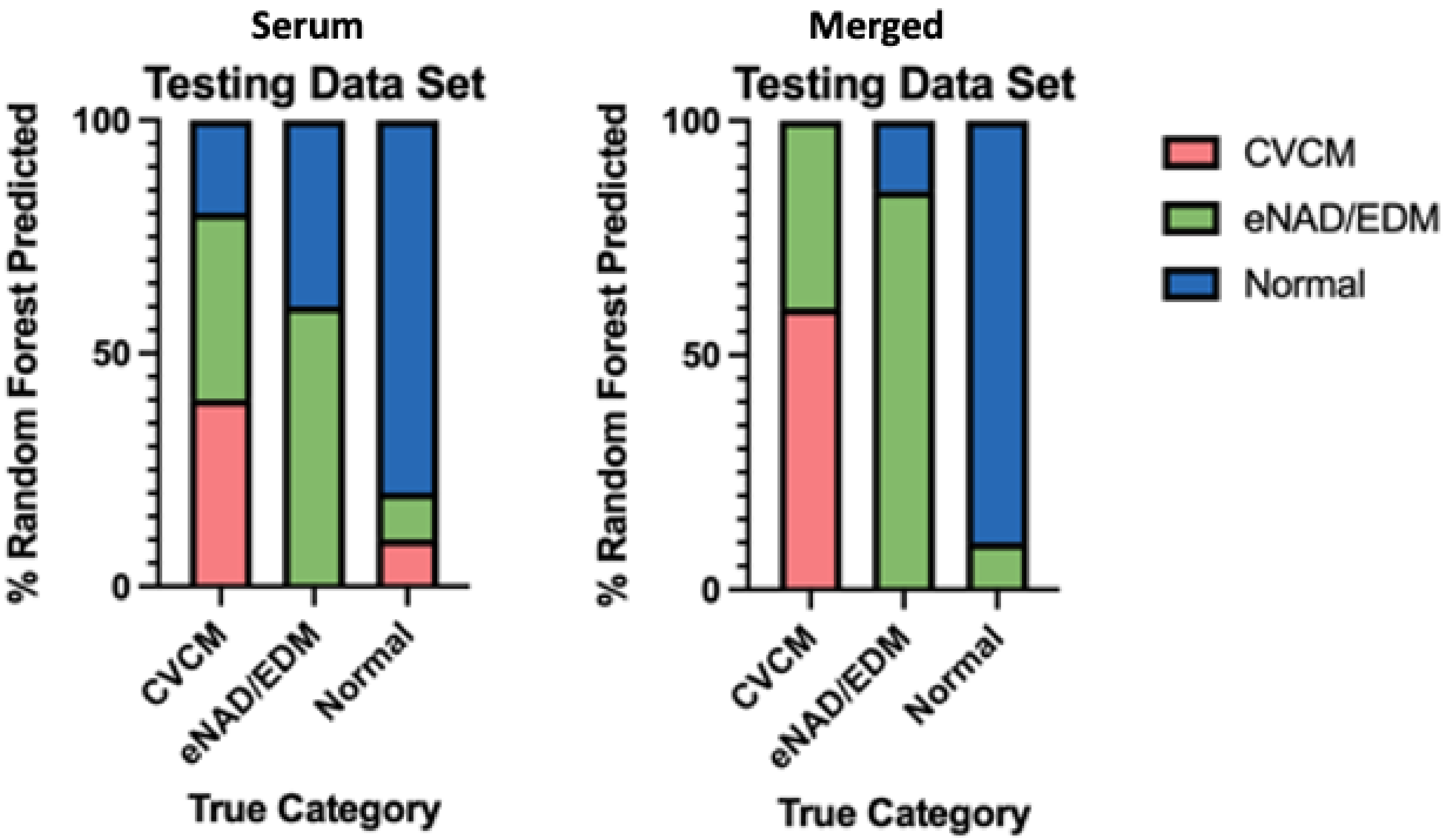

Supplementary Figure 3 Random Forest model testing. Prediction accuracy determined for serum and merged (serum and CSF) data sets. Merged data performed at a similar accuracy to CSF alone, and again did not misclassify any CVCM cases as eNAD/EDM or normal. Serum performed the least well of all three analyses with misclassification occurring in all groups.

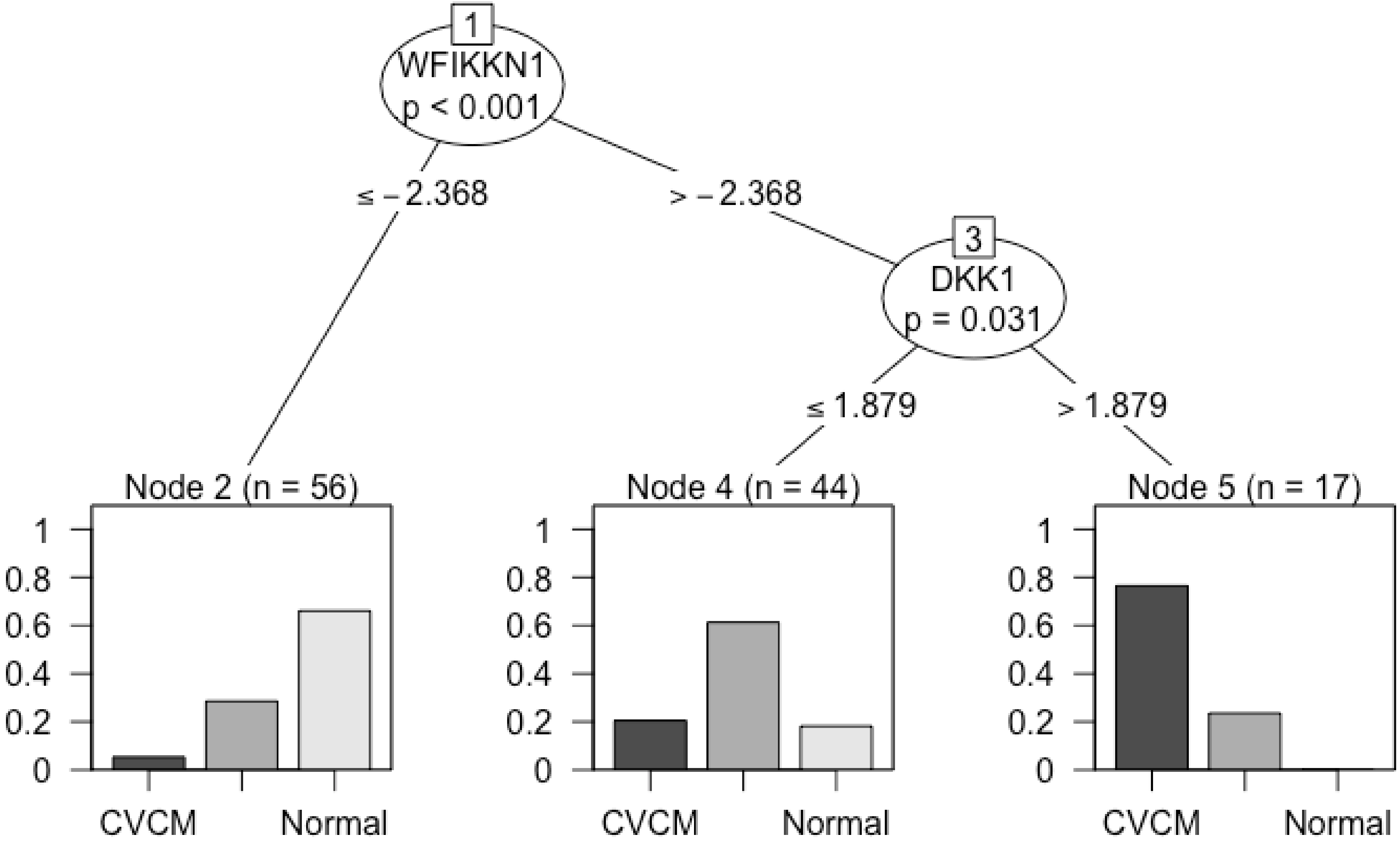

Supplemental Figure 4 Conditional inference model tree for merged serum/CSF. A three- protein model, with CSF RSPO1, CSF NEFL and serum DKK1 had the highest accuracy for prediction of normal horses (85.5%), CVCM (85.5%) and eNAD/EDM (72.7%). Abbreviations: DKK1-Dckkoph 1; NEFL-Neurofilament-light; RSPO1-R-Spondin 1.

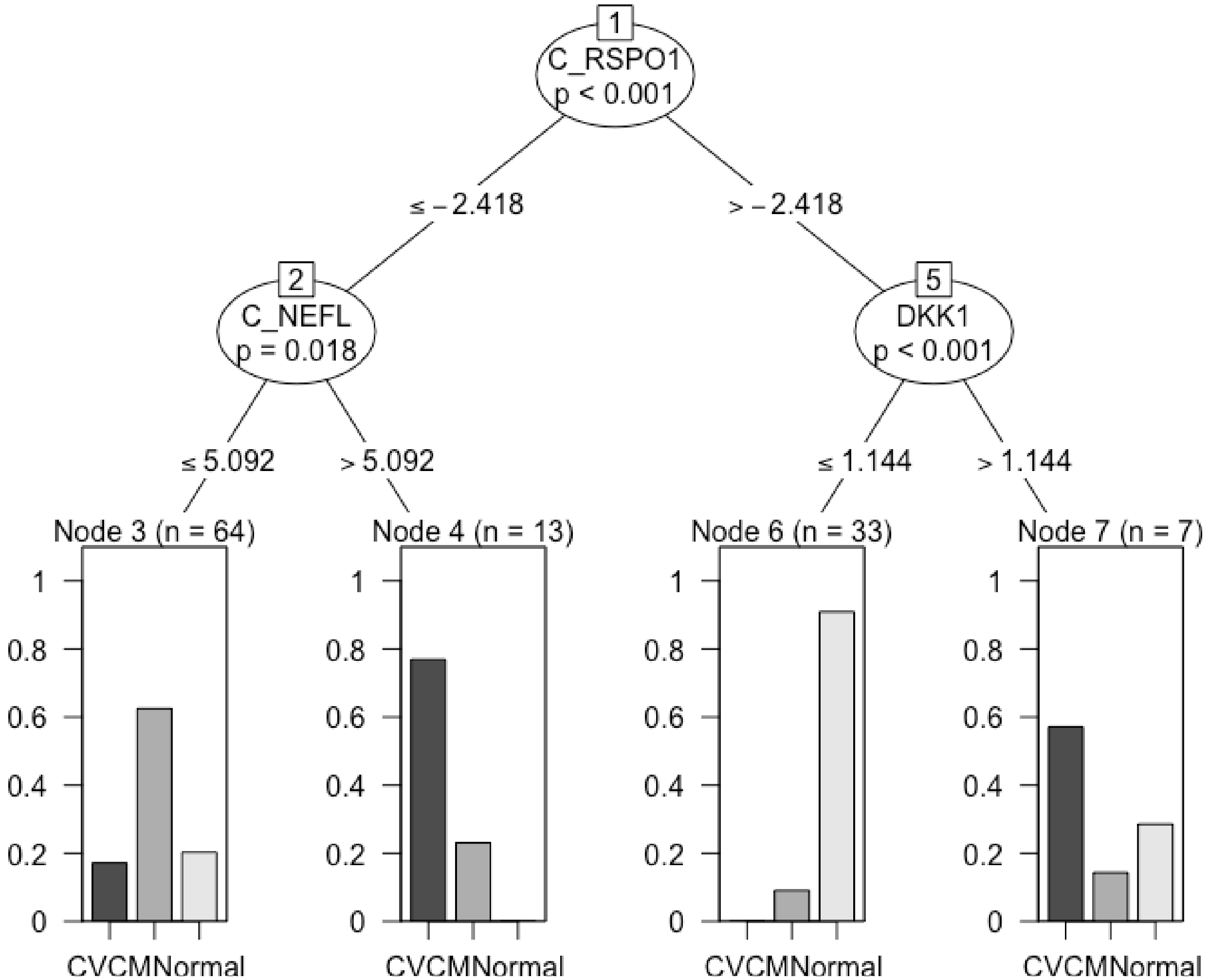

Supplemental Figure 5. Conditional inference model tree for merged serum/CSF. A three- protein model ,with CSF RSPO1, CSF NEFL and serum DKK1 had the highest accuracy for prediction of normal horses (85.5%), CVCM (85.5%) and eNAD/EDM (72.7%). Abbreviations: DKK1-Dckkoph 1; NEFL-Neurofilament-light; RSPO1-R-Spondin 1.
